# Supplementary material for: Impact of Arsenic Stress on the Antioxidant System and Photosystem of Arthrospira platensis
Source: Biology (Basel). 2024 Dec 15;13(12):1049. doi: 10.3390/biology13121049 (PMC11673294; doi:10.3390/biology13121049)
Supplement: Supplementary file 1 [file biology-13-01049-s001.zip › biology-3344913-Supplementary Materials.pdf]

Supplementary Materials:

Table S1: Zarrouk culture medium

Reagents name	Reagents purity	Concentration(g/L)	Manufacturer
$NaHCO_3$	analytical pure	16.8	Aladdin
$NaNO_3$	analytical pure	2.5	Tianjin Beilian
NaCl	analytical pure	1.0	Aladdin
$FeSO_4·7H_2O$	analytical pure	0.01	Macklin
$MgSO_4·7H_2O$	analytical pure	0.2	Macklin
K_2SO_4	analytical pure	1.0	Aladdin
$K_2HPO_4·3H_2O$	analytical pure	0.5	Sigma
$CaCl_2$	analytical pure	0.04	Aladdin

Table S2: The primer sequences

Gene name	primer sequences
16sRNA	Forward primer：AGCTGCTCAGGGTTTAGATGG
	Reverse primer：TGTCCGGGTTTTTGTAGCCA
hoxE	Forward primer：TTCTGGGGTTTGCTGACCTG
	Reverse primer：TGGAGAAGTCCTAGCAGCCT
hoxU	Forward primer：TCGTGTTTCTCCCGTGCATT
	Reverse primer：TTGTCCGACGGGTTCCATTT
hoxF	Forward primer：GCTTTAACTTGCAGACCGCC
	Reverse primer：AATCCGCAATAACGGCCTGA
cydB	Forward primer：TCAGCCGCCGCTTGATAAAT
	Reverse primer：ATCACTGTGGTAACGCCGAT
mgtC	Forward primer：CTCACGGTTAAGGGGTTGAA
	Reverse primer：CTTCATCACCCCGACAAACT

Trxs	Forward primer：GCGATAATTTCTCCGCCCCT<br>Reverse primer：ACTGGACAGCCGACTTTAGC
